# Supplementary material for: Chemical inhibition of the integrated stress response impairs the ubiquitin-proteasome system
Source: Commun Biol. 2024 Oct 8;7:1282. doi: 10.1038/s42003-024-06974-0 (PMC11461528; doi:10.1038/s42003-024-06974-0)

Xu et al "Chemical inhibition of the integrated stress response impairs the ubiquitin-proteasome system"

Supplementary Figures 1-5

Suppl. Fig 1: Induction of ISR by heat shock and ISRIB effect on cells' viability

Suppl. Fig 2: Accumulation of Ub-YFP in response to proteasome inhibition.

Suppl. Fig 3: Proteasome activity and sensitivity to proteasome inhibition.

Suppl. Fig 4: Gating strategy for flow cytometry experiment.

Suppl. Fig 5: Uncropped blots.

Suppl. Fig. 1

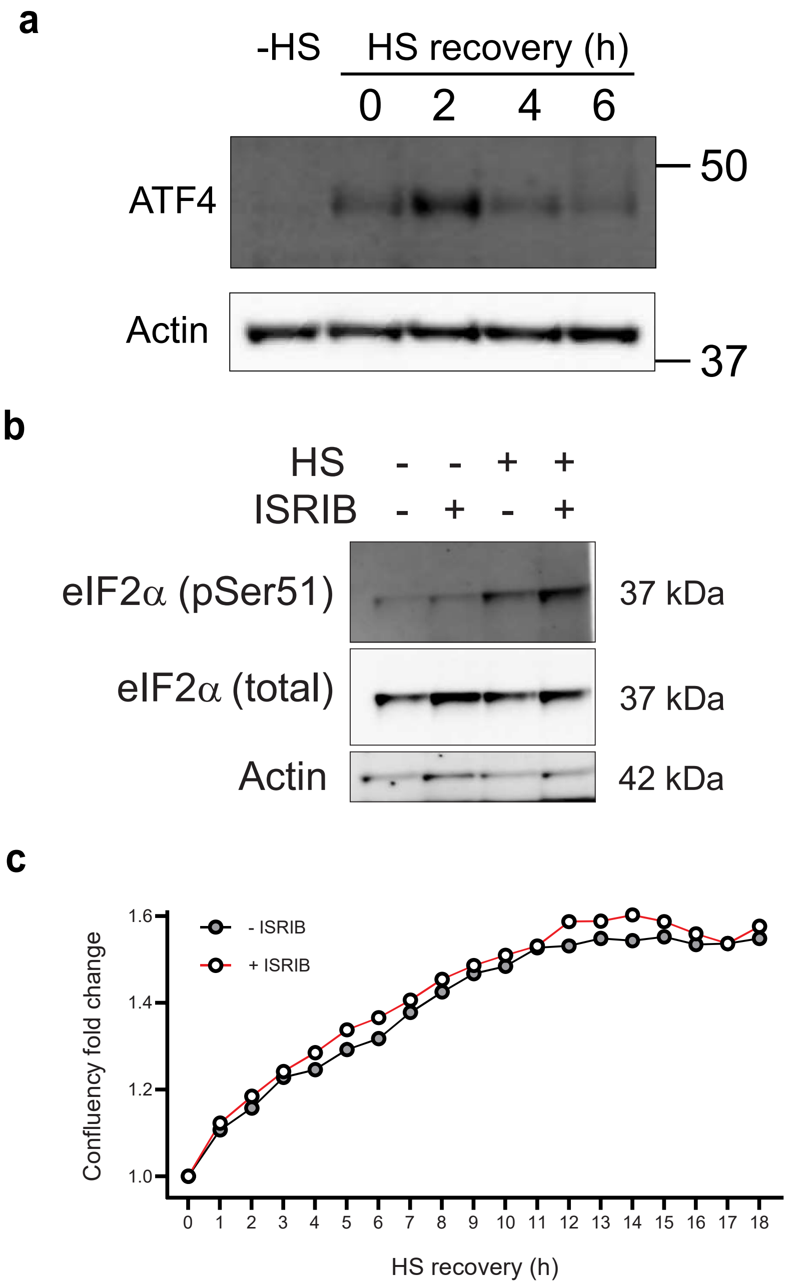

Supplementary Figure 1. Induction of ISR by heat shock and ISRIB effect on cells' viability.
a) MelJuSo were exposed to a 30-minute heat shock at 43°C, to monitor ATF4 levels in the recovery phase (HS recovery). Cells were harvested at the indicated time points after heat shock and a non-heat shocked sample was used as a control reference (-HS).
b) MelJuSo were left untreated (-ISRIB) or pre-treated with ISRIB (+ISRIB) for 30 minutes, followed by either no further treatment (-HS) or exposure to 43°C for 30 minutes (+HS). Cells were harvested right after heat shock and cell lysates were probed for phosphorylated eIF2α (pSer51), total eIF2α and β-actin.
c) Assessing the effects of ISRIB on cell viability upon heat shock. MelJuSo were pre-treated with ISRIB for 30 minutes before a 30-minute heat shock session at 43°C. Cells were then imaged for 18 hours, once every hour, and cells confluency fold change was measured. Each time point and condition was imaged at four different sites, and the graph shows the average confluency of the four images.

Suppl. Fig. 2

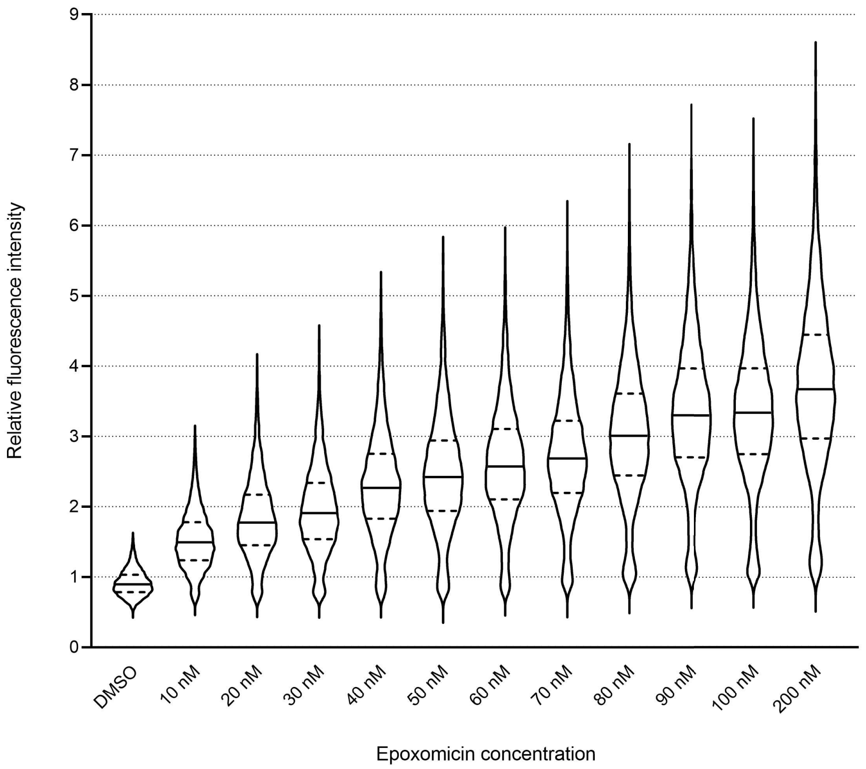

Supplementary Figure 2. Accumulation of Ub-YFP in response to proteasome inhibition. MelJuSo expressing Ub-YFP and mCherry-G3BP1 were treated for 4 hours with increasing concentrations of the proteasome inhibitor epoxomicin (EPX). For each condition, the mean fluorescence intensity of at least 7000 cells was quantified. The frequency and distribution of the relative YFP fluorescence intensities per cell are shown as violin plots. The solid lines in each distribution represent the median, and the dash lines represent the upper and lower interquartile range limits (>7000 cells analysed per condition). One representative experiment out of two.

Suppl. Fig. 3

a

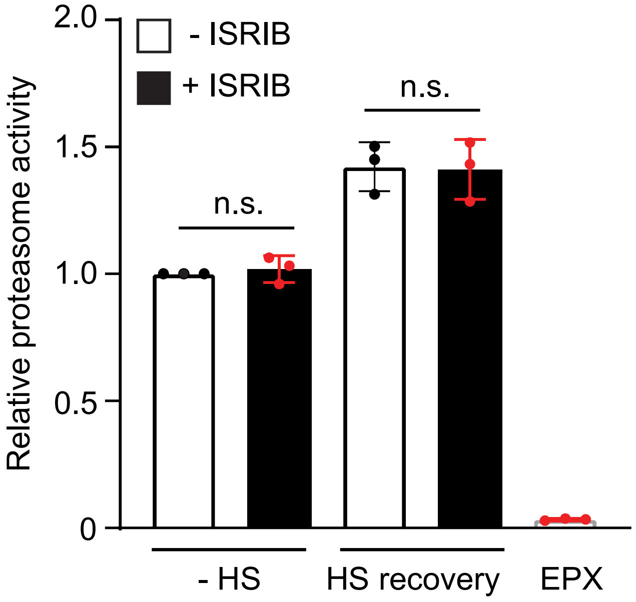

b

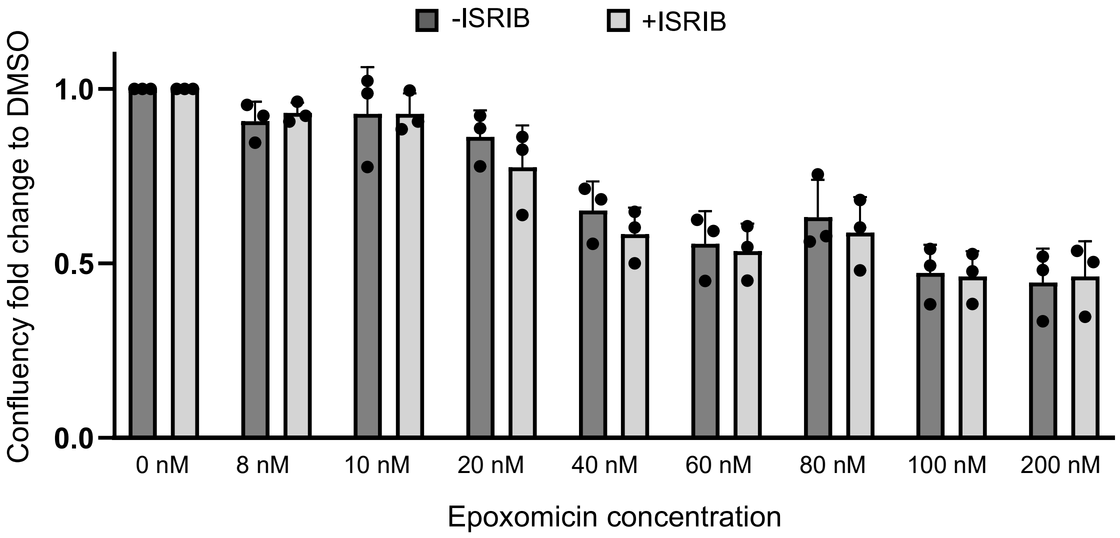

Supplementary Figure 3. Proteasome activity and sensitivity to proteasome inhibition.
a) Proteasome activity in ISRIB-treated cells. MelJuSo expressing Ub-YFP cells were pretreated in the absence (-ISRIB) or presence (+ISRIB) of ISRIB for 30 minutes and followed by either left untreated (-HS), exposed to 43°C for 30 minutes and followed for 4 hours after heat shock (HS recovery). The chymotrypsin activity (β5 subunit) of the proteasome was detected by following conversion of the fluorogenic Suc-LLVY-AMC substrate over 1 hour. As a control, 100 nM proteasome inhibitor epoxomicin (EPX) was added to the reaction mixture to inhibit proteasome activity. Data represents the mean ± SD. (three independent experiments, n.s.: not significant).
b) Analysis of ISRIB effect on cells sensitivity to proteasome inhibition, during heat shock recovery. MelJuSo were pre-treated without (-ISRIB) or with ISRIB (+ISRIB) for 30 minutes. Cells were then exposed to a 30-minute heat shock at 43°C, after which they were treated with increasing concentrations of epoxomicin and let recover for 18 hours at 37°C. The bar plot shows the average confluency fold change for each treatment concentration, normalized to the corresponding untreated sample (with or without ISRIB treatment). Data were acquired from 3 independent experiments.

Suppl. Fig. 4 Gating strategy

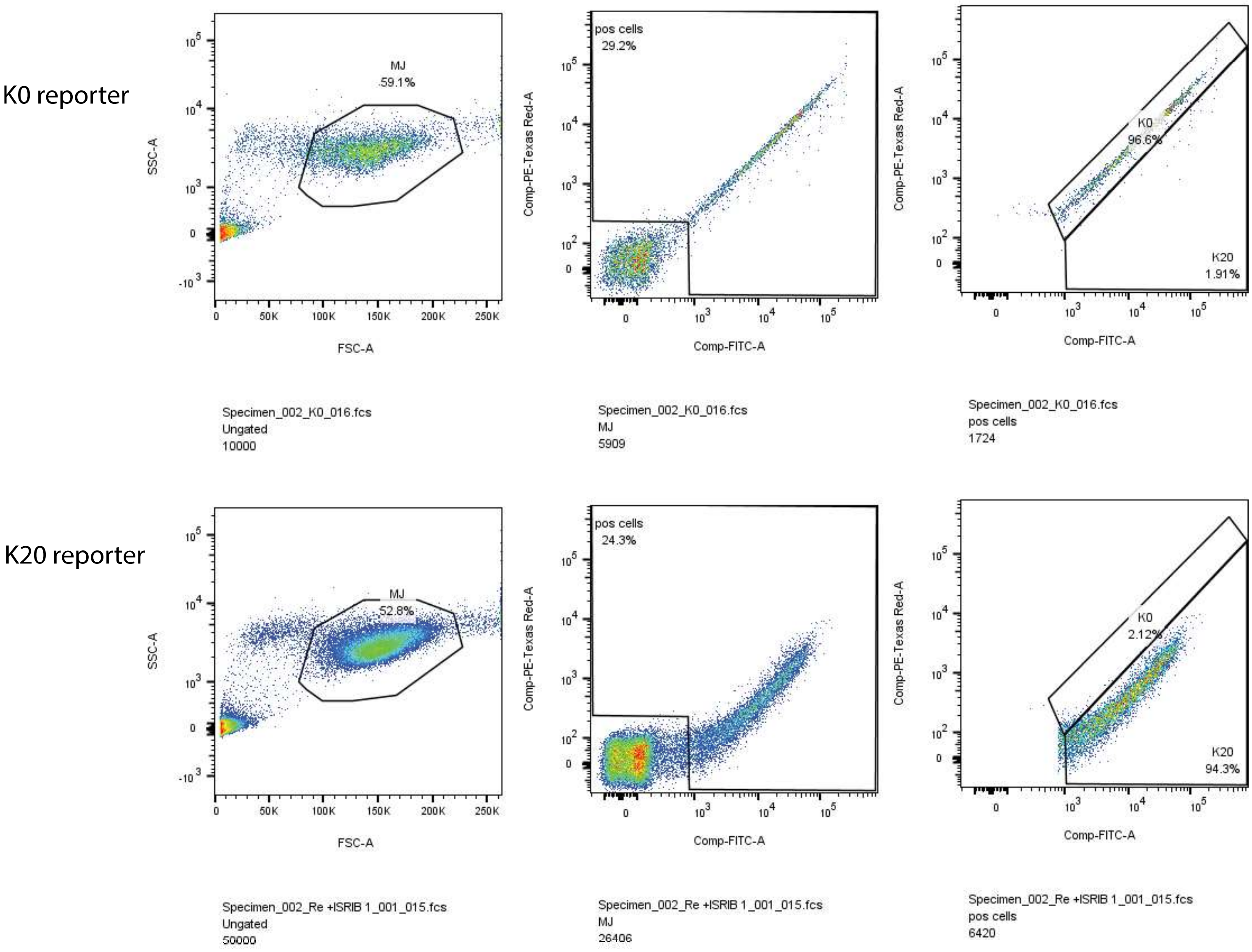

Supplementary Figure 4. Gating strategy for flow cytometry experiment.
The gating strategy for the flow cytometry experiment with K0 and K20 reporter shown in Fig. 5a.

Suppl. Fig. 5

Fig 1d

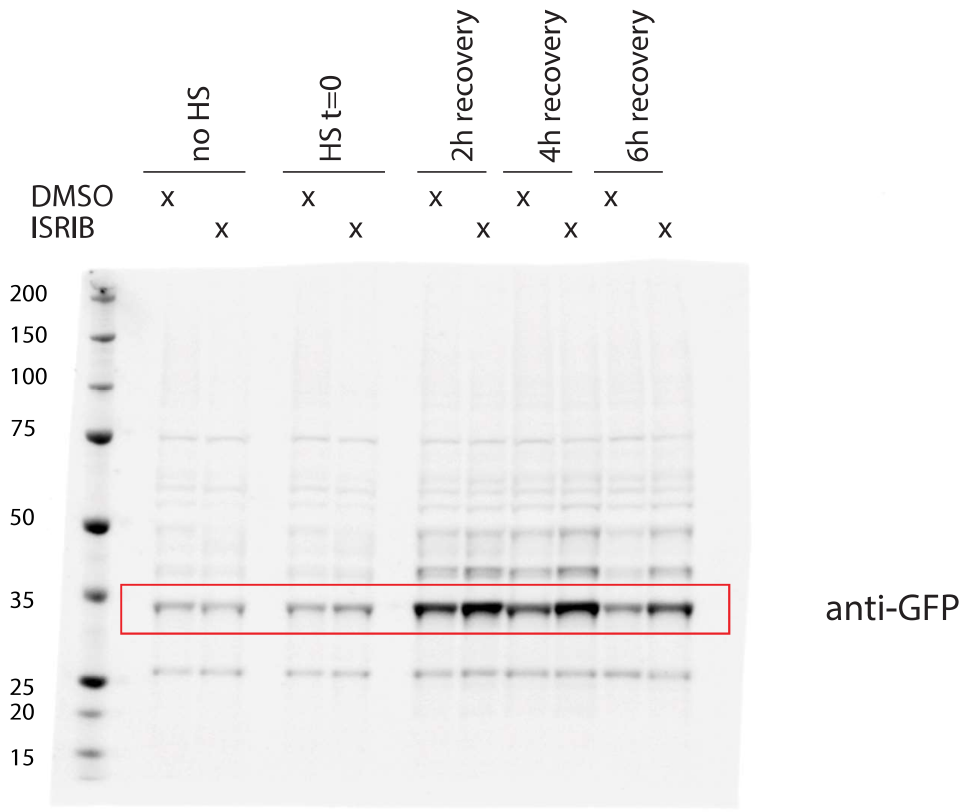

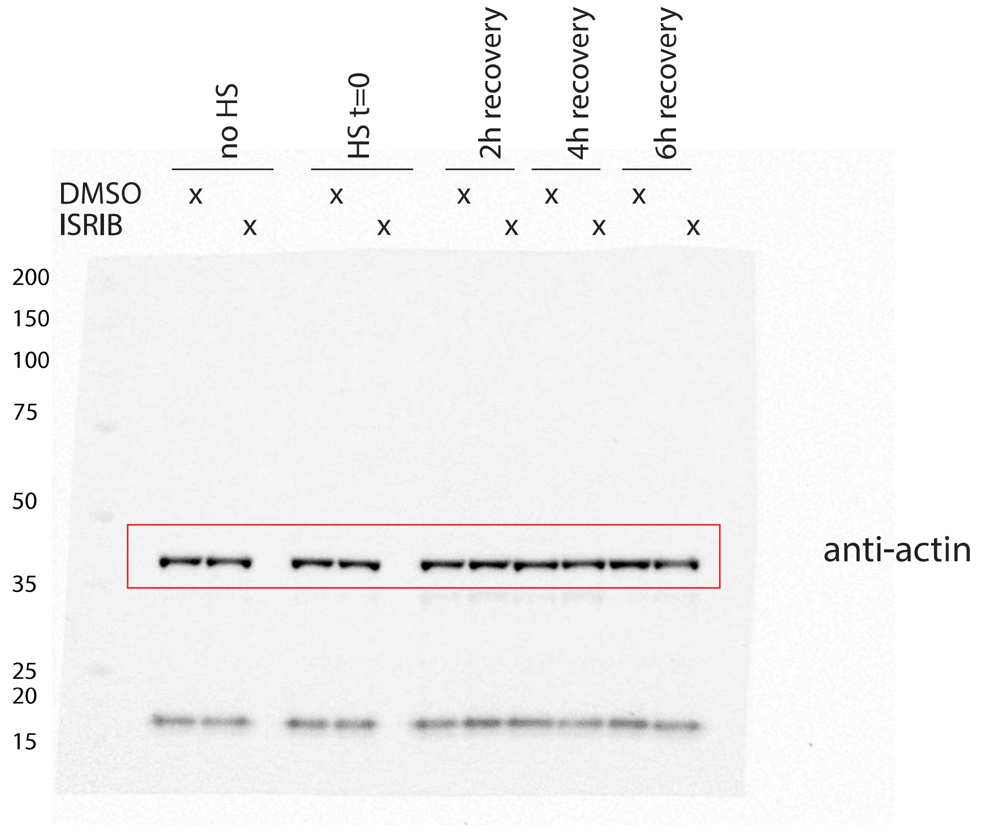

Fig 1e

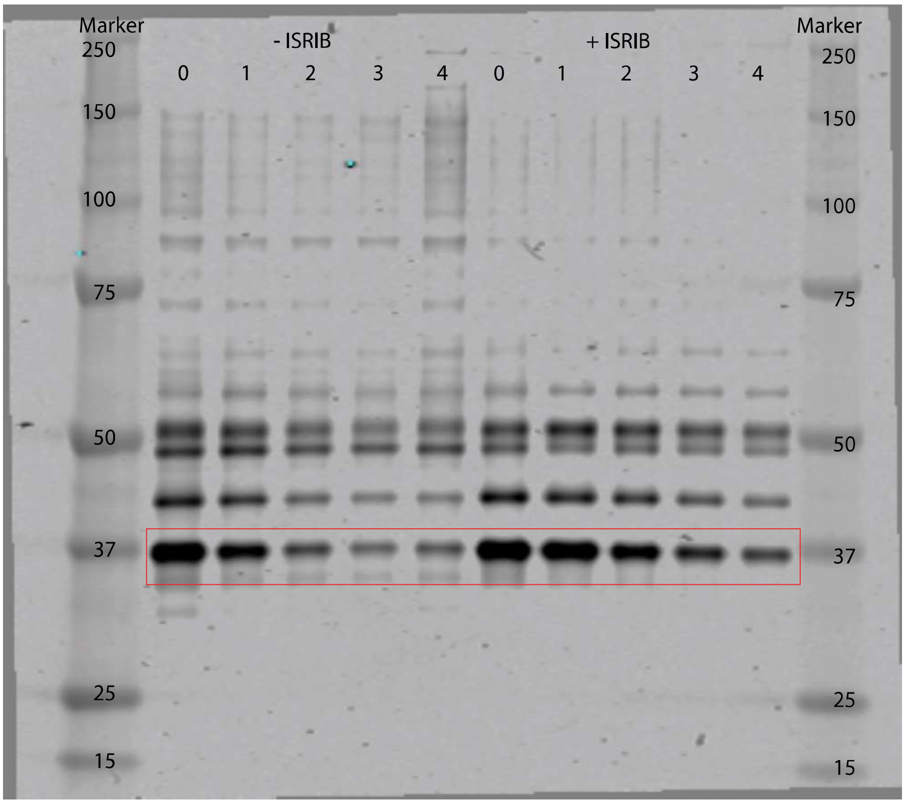

Marker
250
150
100
75
50
37
25
15
- ISRIB
0 1 2 3 4
+ ISRIB
0 1 2 3 4
Marker
250
150
100
75
50
37
25
15

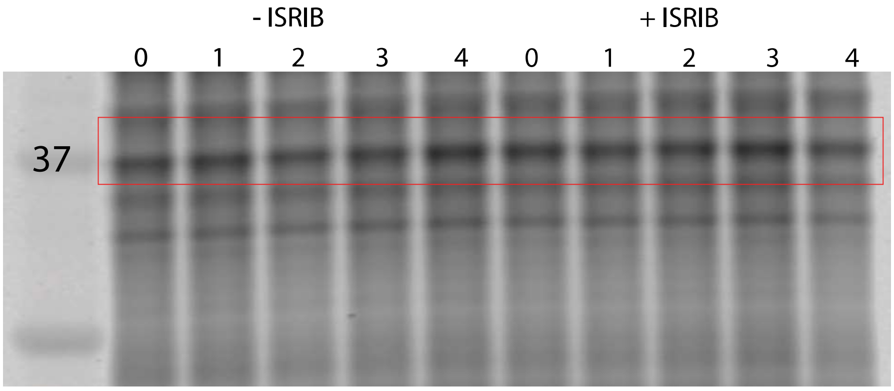

- ISRIB
+ ISRIB
0 1 2 3 4 0 1 2 3 4
37

Figure 4

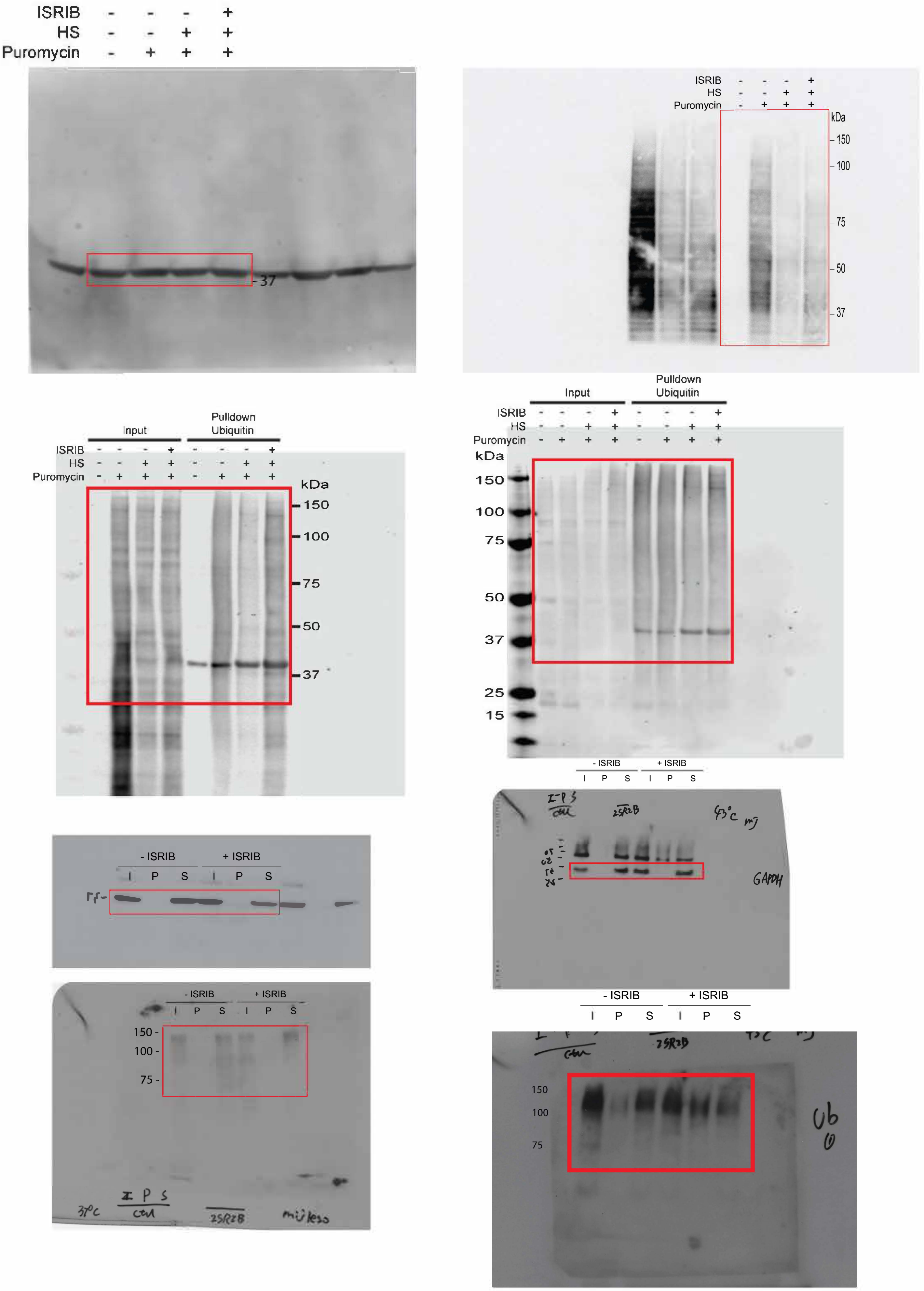

ISRIB - - - +
HS - - + +
Puromycin - + + +
37
ISRIB - - - +
HS - - + +
Puromycin - + + +
kDa
150
100
75
50
37
Input
Pulldown Ubiquitin
ISRIB - - - + - - - +
HS - - + + - - + +
Puromycin - + + + - + + +
kDa
150
100
75
50
37
Input
Pulldown Ubiquitin
ISRIB - - - + - - - +
HS - - + + - - + +
Puromycin - + + + - + + +
kDa
150
100
75
50
37
25
15
- ISRIB
+ ISRIB
I P S I P S
GAPDH
- ISRIB
+ ISRIB
I P S I P S
- ISRIB
+ ISRIB
I P S I P S
150 -
100 -
75 -
- ISRIB
+ ISRIB
I P S I P S
150
100
75
Ub

Suppl. Fig. 1a

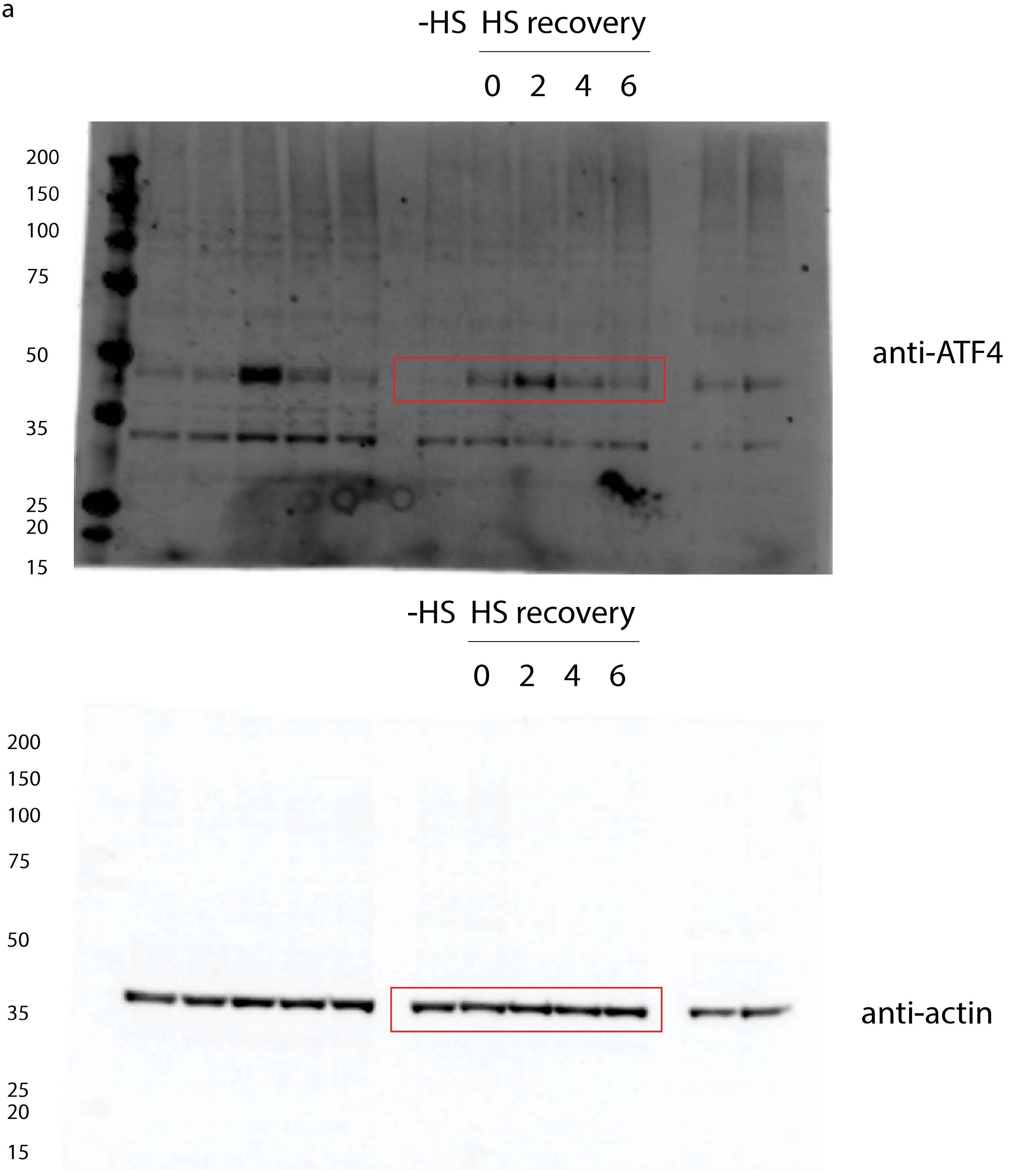

Suppl. Fig. 1b

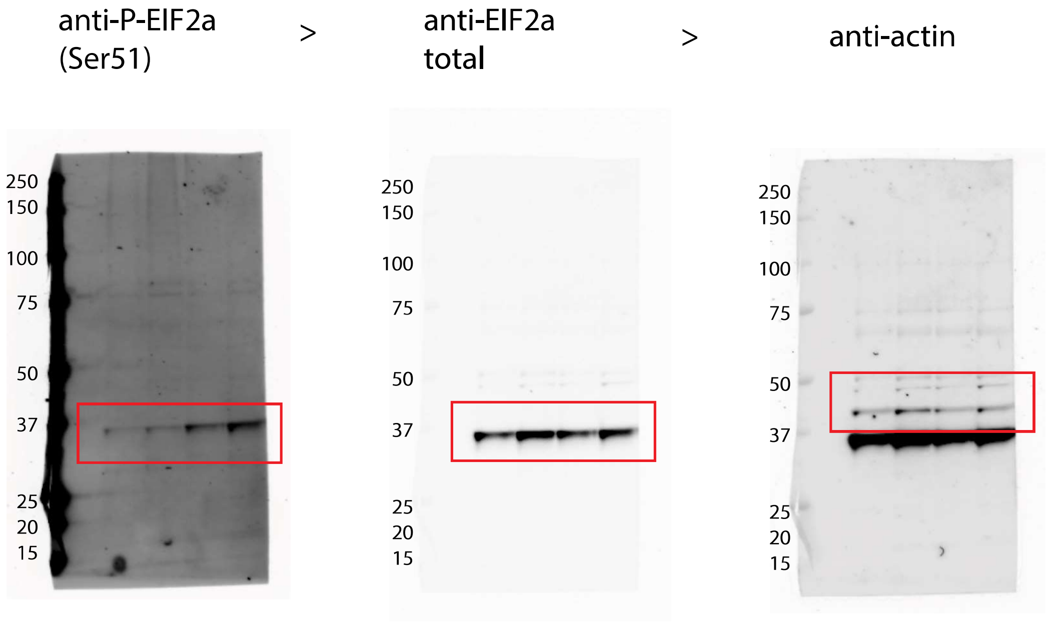

Supplement: Supplementary file 2 — Supplementary Information [file 42003_2024_6974_MOESM2_ESM.pdf]
