# Supplementary material for: Chemical inhibition of the integrated stress response impairs the ubiquitin-proteasome system
Source: Commun Biol. 2024 Oct 8;7:1282. doi: 10.1038/s42003-024-06974-0 (PMC11461528; doi:10.1038/s42003-024-06974-0)
Supplement: Supplementary file 5 — Reporting Summary [file 42003_2024_6974_MOESM5_ESM.pdf]

nature portfolio

Corresponding author(s): Nico Dantuma

Last updated by author(s): 28/08/2024

Reporting Summary

Nature Portfolio wishes to improve the reproducibility of the work that we publish. This form provides structure for consistency and transparency in reporting. For further information on Nature Portfolio policies, see our Editorial Policies and the Editorial Policy Checklist.

Statistics

For all statistical analyses, confirm that the following items are present in the figure legend, table legend, main text, or Methods section.

n/a	Confirmed	
☐	☒	The exact sample size (*n*) for each experimental group/condition, given as a discrete number and unit of measurement
☒	☐	A statement on whether measurements were taken from distinct samples or whether the same sample was measured repeatedly
☐	☒	The statistical test(s) used AND whether they are one- or two-sided<br>*Only common tests should be described solely by name; describe more complex techniques in the Methods section.*
☒	☐	A description of all covariates tested
☐	☒	A description of any assumptions or corrections, such as tests of normality and adjustment for multiple comparisons
☐	☒	A full description of the statistical parameters including central tendency (e.g. means) or other basic estimates (e.g. regression coefficient) AND variation (e.g. standard deviation) or associated estimates of uncertainty (e.g. confidence intervals)
☒	☐	For null hypothesis testing, the test statistic (e.g. *F*, *t*, *r*) with confidence intervals, effect sizes, degrees of freedom and *P* value noted<br>*Give P values as exact values whenever suitable.*
☒	☐	For Bayesian analysis, information on the choice of priors and Markov chain Monte Carlo settings
☒	☐	For hierarchical and complex designs, identification of the appropriate level for tests and full reporting of outcomes
☒	☐	Estimates of effect sizes (e.g. Cohen's *d*, Pearson's *r*), indicating how they were calculated

Our web collection on statistics for biologists contains articles on many of the points above.

Software and code

Policy information about availability of computer code

Data collection	N.A.
Data analysis	ImageJ, CellProfiler and IncuCyte for microscopy, Flowjo for flow cytometry

For manuscripts utilizing custom algorithms or software that are central to the research but not yet described in published literature, software must be made available to editors and reviewers. We strongly encourage code deposition in a community repository (e.g. GitHub). See the Nature Portfolio guidelines for submitting code & software for further information.

Data

Policy information about availability of data

All manuscripts must include a data availability statement. This statement should provide the following information, where applicable:

- Accession codes, unique identifiers, or web links for publicly available datasets
- A description of any restrictions on data availability
- For clinical datasets or third party data, please ensure that the statement adheres to our policy

Included in manuscript

Human research participants

Policy information about studies involving human research participants and Sex and Gender in Research.

Reporting on sex and gender	NA
Population characteristics	NA
Recruitment	NA
Ethics oversight	NA

Note that full information on the approval of the study protocol must also be provided in the manuscript.

Field-specific reporting

Please select the one below that is the best fit for your research. If you are not sure, read the appropriate sections before making your selection.

☒ Life sciences ☐ Behavioural & social sciences ☐ Ecological, evolutionary & environmental sciences

For a reference copy of the document with all sections, see nature.com/documents/nr-reporting-summary-flat.pdf

Life sciences study design

All studies must disclose on these points even when the disclosure is negative.

Sample size	Quantifications are from three independent experiments or the indicated number of cells.
Data exclusions	No data were excluded.
Replication	All experiments have been replicated at least three times.
Randomization	Samples were handled as much as possible randomly.
Blinding	The researchers performing the experiments were not blinded for the sample identities.

Reporting for specific materials, systems and methods

We require information from authors about some types of materials, experimental systems and methods used in many studies. Here, indicate whether each material, system or method listed is relevant to your study. If you are not sure if a list item applies to your research, read the appropriate section before selecting a response.

Materials & experimental systems

n/a	Involved in the study	
☐	☒	Antibodies
☐	☒	Eukaryotic cell lines
☒	☐	Palaeontology and archaeology
☒	☐	Animals and other organisms
☒	☐	Clinical data
☒	☐	Dual use research of concern

Methods

n/a	Involved in the study	
☒	☐	ChIP-seq
☐	☒	Flow cytometry
☒	☐	MRI-based neuroimaging

Antibodies

Antibodies used	G3BP1, TIA1, beta-actin, GAPDH, puromycin, GFP, eIF2alfa(pSer51), eIF2alfa, ATF4, ubiquitin
Validation	All antibodies are commercially available. Suppliers are mentioned in manuscript. Antibodies have not been independently validated by authors.

Eukaryotic cell lines

Policy information about cell lines and Sex and Gender in Research

Cell line source(s)	Human melanoma cell line MelJuSo
Authentication	Cell line has not been recently authenticated.
Mycoplasma contamination	Cells are frequently PCR tested on mycoplasma.
Commonly misidentified lines (See ICLAC register)	NA

Flow Cytometry

Plots

Confirm that:

☒ The axis labels state the marker and fluorochrome used (e.g. CD4-FITC).

☒ The axis scales are clearly visible. Include numbers along axes only for bottom left plot of group (a 'group' is an analysis of identical markers).

☒ All plots are contour plots with outliers or pseudocolor plots.

☒ A numerical value for number of cells or percentage (with statistics) is provided.

Methodology

Sample preparation	Cells have been collected in eppis after trypsinization from the plate, spin down at 2000rpm for 5min, washed once in PBS, spin down again for 5min at 2000rpm and diluted in 500ul PBS for flow cytometry analysis. Cells were kept on ice until analysis.
Instrument	BD® LSR II SORP Flow Cytometer
Software	Data were collected using BD Diva software. Data were analysed using FlowJo Treestar v10
Cell population abundance	50 000 cells were recorded from each sample with K20 reporter. 10 000 cells were recorded for the K0 reporter. Double positive reporter positive cells were analysed after compensation with the single colors using BD Divas compensation setup function. Around 30-60% of the cells were positive for the double reporter.
Gating strategy	We gated first for living cells based on SSC-A vs FSC-A. We gated then for single cells based on FSC-A to FSC-W. We then gated the positive cells based on compensated Texas-Red-A vs Compensated FITC-A. From all the positive cells, we gated around the K0 double reporter and plotted % cells of the K0 gate as read-through.

☒ Tick this box to confirm that a figure exemplifying the gating strategy is provided in the Supplementary Information.
